# Presentation of severe brucellosis in 5-year-old boy - challenges and results

**DOI:** 10.1186/s12879-023-08138-7

**Published:** 2023-03-17

**Authors:** Yulia Dinikina, Uliana Tsoy, Andrey Dyachkov, Roman Grozov, Aleksandr Mushkin, Vyacheslav Zorin, Irina Nikitina, Elena Grineva

**Affiliations:** 1 Almazov National Medical Research Centre, Saint-Petersburg, Russia; 2 First Pavlov State Medical University, Saint-Petersburg, Russia; 3 Science-Research institute of Phthisiopulmonology Ministry of health of Russian Federation, Saint-Petersburg, Russia; 4 H. Turner National Medical Research Center for Сhildren’s Orthopedics and Trauma Surgery, Saint-Petersburg, Russia

**Keywords:** Brucellosis, Child, SIADH, Hyponatremia, Bone fractures, Lymphadenopathy

## Abstract

Brucellosis is highly contagious zoonotic bacterial disease caused by gram-negative genus. It has a wide spectrum of clinical manifestations and due to variety and nonspecificity of clinical signs the diagnostics can be very complicated. We present a clinical case of severe chronic brucellosis in a 5-years old boy with long-term course of disease and multiorgan involvement. A different complication of brucellosis including severe syndrome of inappropriate ADH secretion (SIADH) are discussed. Despite severe course of disease patient achieved significant clinical improvement due to multidisciplinary approach and optimal etiotropic and pathogenetic treatment.

**Supplementary Information:**

The online version contains supplementary material available at 10.1186/s12879-023-08138-7.

## Introduction

Brucellosis is an endemic zoonotic disease with a reported average people morbidity rate as 0,24 per 100 000 in Russia and as 0,1 – in children under 17. The largest prevalence of brucellosis among people was registered in the North-Caucasian (77,1%) and the Southern Federal District (13,5%), but during the period of 2010–2020 more than 5000 potentially hazardous areas as regards bovine, sheep and goats’ brucellosis were registered in the Russian Federation [1]. Brucella spp. are small, non-spore-forming, nonfermenting, facultative intracellular, Gram-negative coccobacilli [2]. *Brucella melitensis, Brucella abortus* and *Brucella suis* are known to cause most cases of human disease [3, 4]. The most common route of transmission to humans is a direct contact with the secretion of infected animals or their products through conjunctiva or abrasion in the skin, consumption of raw milk and dairy products (soft cheese, butter, cream) and air borne transmission of aerosolized materials [2, 4].

Brucellosis exhibits variable clinical and laboratory characteristics that can mimic other infectious and non-infectious conditions [2, 5]. In spite of existence of strict laboratory diagnostic criteria, such factors as the phase of disease (acute, chronic or relapse), variety of clinical presentations and history of antibacterial therapy, may complicate diagnosis verification [6, 7]. SIADH with hyponatremia, being very rare disorder in children, is described in association with Brucellosis and can be used as a hint for clinical diagnosis [8, 9].

Management of brucellosis include prolonged dual or triple antimicrobial regimen with a limited number of antibiotics according to the system involved and patient’s age. [2, 4, 10, 11]. Usually, patients show improvement within 3–7 days after starting appropriate therapy [1, 2], but symptomatic supportive care can be necessary [2, 4, 8, 11, 12]. Even when patients are adequately treated, relapses of the disease, usually milder than the initial episode, as well as progression to chronic phase of infection may occur [2]. Among treated patients the prognosis is benign, but 3 to 9% will have a relapse in the first year following therapy [4]. Early diagnostics and appropriate treatment strategies will define brucellosis induced sequelae, disability rate and associated mortality [11].

We describe an extremely rare case of late diagnosis of severe chronic brucellosis in a child whose multiorgan involvement mislead to many alternative diagnosis and cascade of diagnostic procedures. Multidisciplinary approach made it possible to confirm diagnoses and start adequate treatment with a good outcome.

## Clinical case description

A 5-years old Caucasian boy was admitted to Almazov National Medical Centre, St Petersburg, Russia with complaints on fatigue, subfebrile fever, extreme weight loss, periodic abdominal pain, diarrhea, generalized lymphadenopathy and recurrent seizures for diagnosis verification with a suspicion on neuroendocrine tumor.

**Fig. 1:**
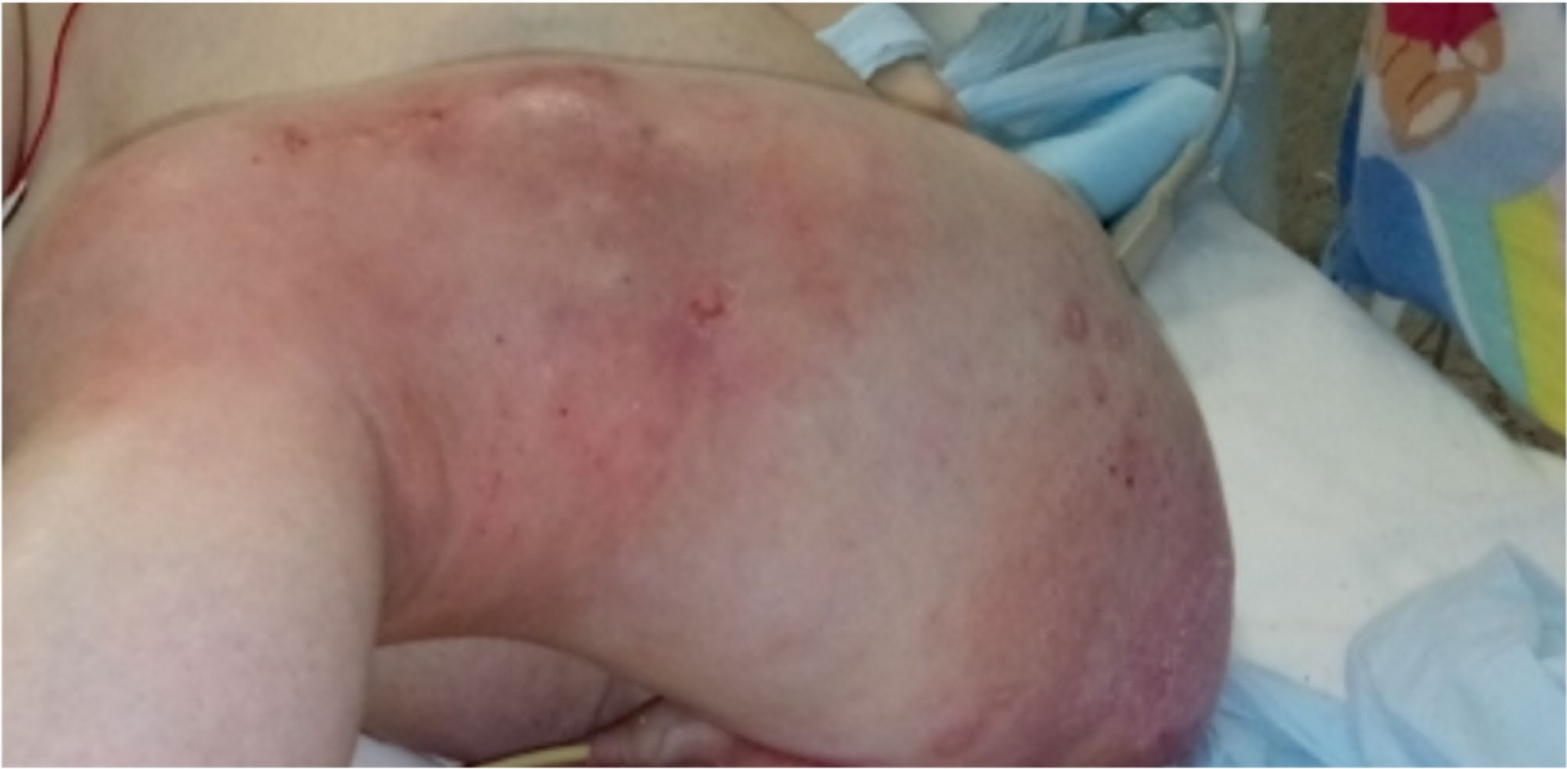
Visible femur fracture with diffuse hyperemia and soft tissue infiltration due to infectious complications

The boy was born in healthy family and there were no abnormalities mentioned in postnatal and early childhood period. At time of admission duration of disease was estimated to be more than 18 months. His first clinical symptoms included periodic febrile fever, polydipsia, excessive sweatiness and diuresis after the boy had spent several weeks in his grandmother’s village where they used home-made dairies (milk, cheese) and meat from the nearest private farm. Complete blood count revealed moderate leukocytosis with eosinophilia. Because there weren’t serious somatic status deterioration and non-permanent symptoms a boy received no treatment. Three months later parents noticed weight loss, persistent abdominal pain with recurrent vomiting and diarrhea. His constitutional symptoms persisted along with development of arthralgia and myalgia, electrolyte disorders (hyponatremia, hypocalcemia), spontaneous bone fractures (both femurs). Serology for brucellosis was performed once with nondirect agglutination test which was negative. During subsequent repeated hospitalizations in regional clinics despite profound examination for excluding infectious (borreliosis, toxoplasmosis, yersiniosis, infectious mononucleosis, parasitic infections) and noninfectious (tumors, endocrine disorders, enzymopathies) conditions, the diagnosis wasn’t verified. Due to severe uncontrolled progressive hyponatremia patient required ICU admission where he received vasopressin receptor antagonist (tolvaptan) in order to control sodium level with positive effect. Also, to control a diverse clinical syndromes combined symptomatic therapy was prescribed, including antihypertensive and antiarrhythmic drugs, calcium and vitamin D supplementations, repeated courses of systemic antibacterial and antifungal therapy for recurrent septic-like episodes associated with soft tissue infections (Fig. 1) and subsequent osteomyelitis. For precise neuroendocrine tumor search the patient was sent to our clinic.

**Fig. 2:**
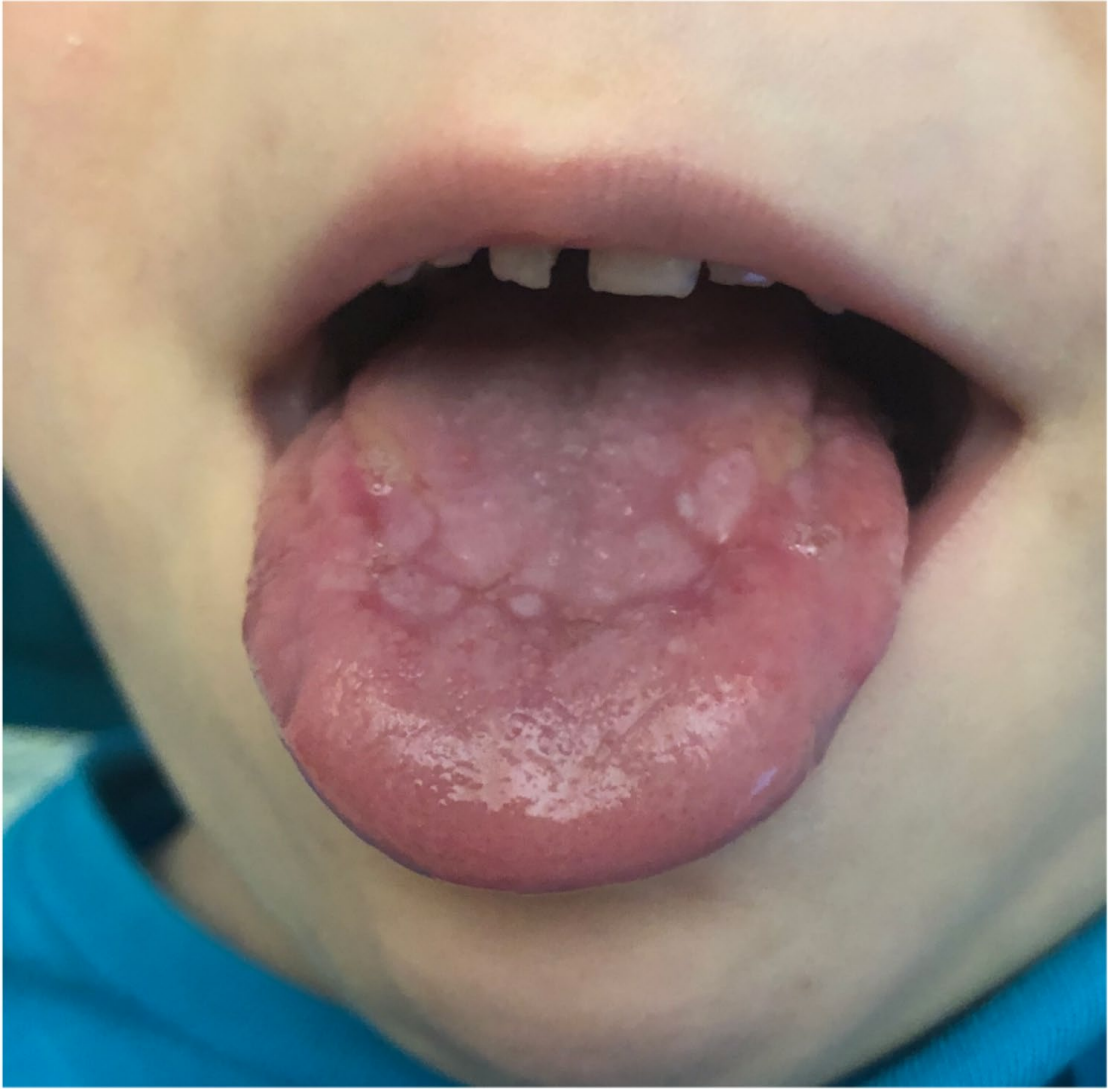
Desquamative glossitis. Ring-formed patterns and erythematous stains

**Fig. 3:**
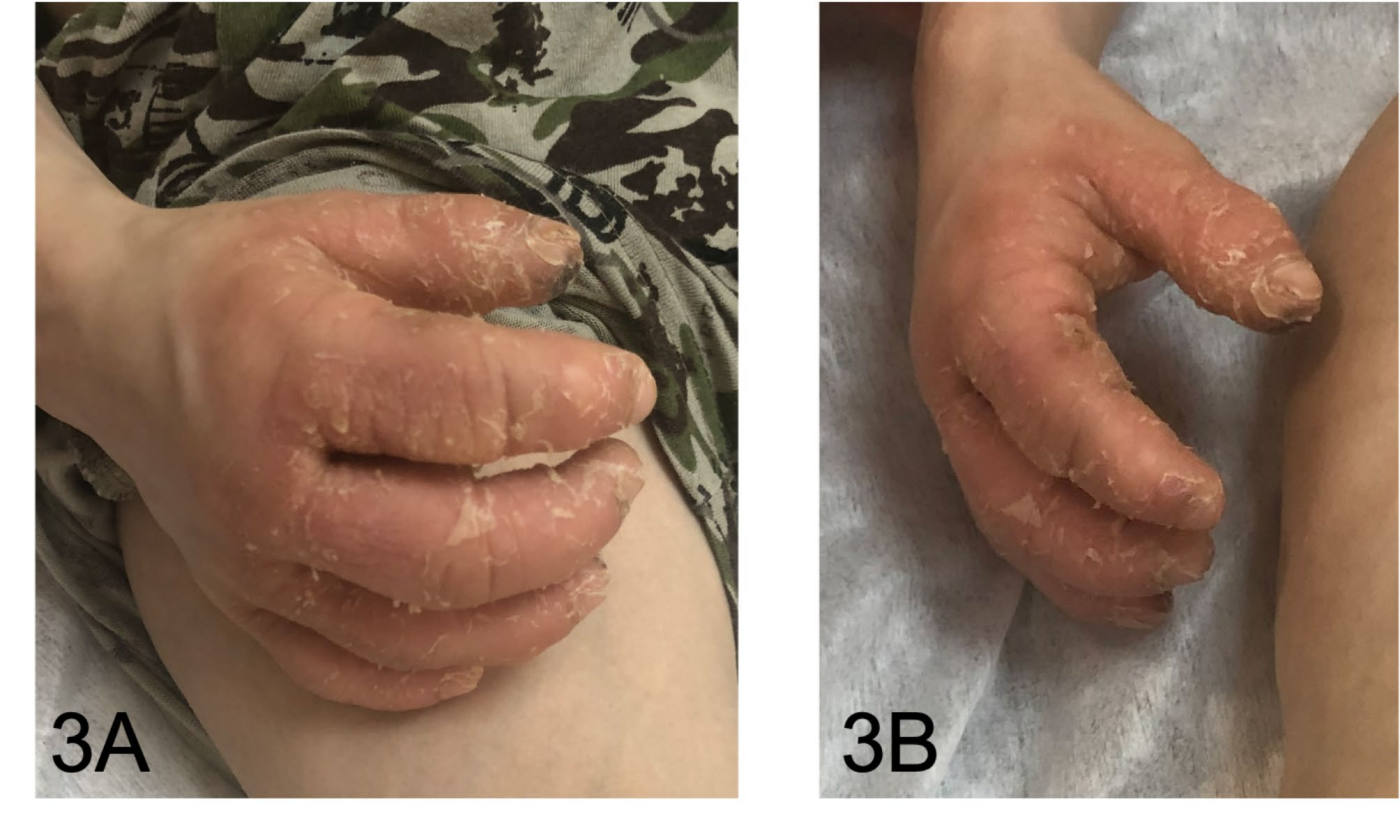
A, B. Enlarged hyperemic hands with lamellar ichthyosis

**Fig. 4:**
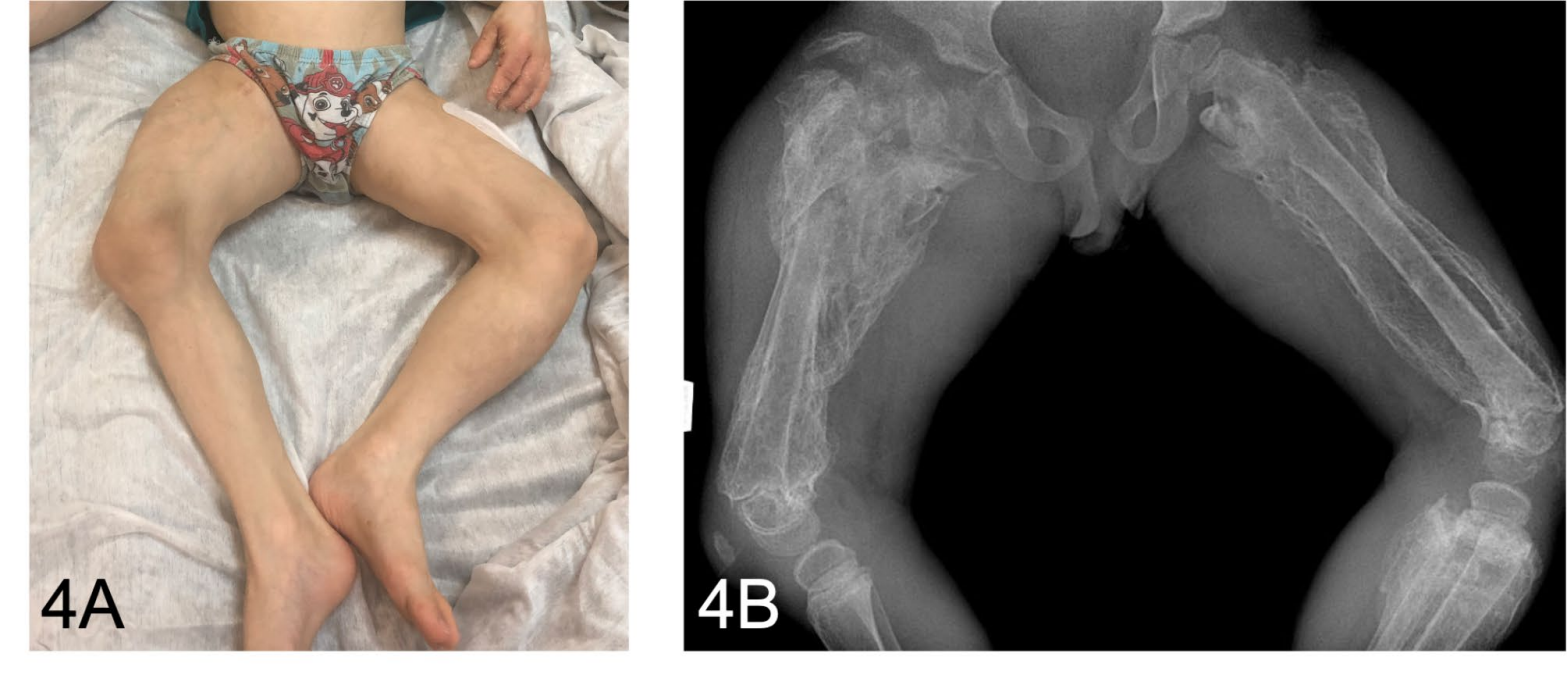
A. Photo of patient’s legs, demonstrating impossibility of full leg extension due to knee-joint contractures. B. X-ray of right and left femurs, demonstrating subperiosteal and calcified hematoma, old fractures of the right femur, osteopenia, hip dysplasia. Pseudoarthrosis with varus of the neck of the right femur with paraossal ossification

The first physical examination in our hospital revealed severe asthenia, limited mobility within the bed, sweatiness, glossitis (Fig. 2), edematous hyperemic hands with ichthyosis (Fig. 3A and B), generalized lymphadenopathy (cervical, axillary, abdominal, inguinal) and hepatosplenomegaly. Deformity of the hips and shins with the formation of rough contractures was noted: flexion-diverting in the hip joints, flexion in the knee and ankle joints. According to X-ray data periosteal ossification was observed throughout the femoral and proximal tibia and pseudoarthrosis with varus of the right femoral neck with periosteal ossification **(**Fig. 4A and B**)**. After admission to our clinic a boy developed stammering, sleeping abnormalities (superficial sleep), recurrent attacks of aggression, intense hand itchiness, painful spontaneous muscular contractions (**Video 1**), tachycardia (140–160 beats per minute) and hypertension (140/100 mmHg). Moreover, patient had diarrhea and during each defecation severe rectal prolapse occurred. Laboratory examination revealed anemia grade 2, electrolyte disorders (hyponatremia with medium serum sodium level 130mEq/L, hypokalemia gr2-3, hypocalcemia gr 3–4), presence of serum hypoosmolarity (< 275mOsm/L) with normal dietary salt intake, hyperexpression of IgE (5313 IU/ml), dyslipidemia, low levels of albumin, Fe, Zn, vitamin D, high level of thyroid-stimulating hormone with low level of free thyroxine, which persisted in spite of substitute therapy. According to immunophenotyping there was decreased proliferation of CD3+, TH-cells, increased CD19 + and abnormal ratio CD4+/CD8+, hyperexpression of general IgG, IgM and IgE.

During the whole period of examination (including anamnestic data) we identified several predominant syndromes, presented in Table 1 (Table 1). We provided instrumental and imaging diagnostics including total body MRI, CT and X-ray of involved bones, 18 F-DOFA and 68Ga-DOTA-NOC PET CT, precise kidneys, thyroid and parathyroid glands ultrasound, esophagogastroduodenoscopy and colonoscopy, echocardiogram, ECG-monitoring and electroencephalogram. Biopsy of involved skin, muscles, lymph nodes and bone marrow cytology were provided. According to received results there were no signs of tumor, systemic rheumatological and endocrine diseases. To exclude enzymopathies immunoinflammatory, syndrome and unknown pathogenic genetic disorders whole exome sequencing was made and no significant variants were revealed.

**Table 1:** Predominant syndromes identified in a patient according to clinical data, instrumental and laboratory evaluation

Presentation		Clinical signs
Intoxication syndrome		Subfebrile fever, fatigue, weight loss, excess sweatiness, tachycardia
Toxico-allergic dermatitis		Dermatitis (hands, perianal region), itches, hyper- IgE-emia
Generalized lymphadenopathy		Cervical, axillary, abdominal, inguinal
Secondary endocrine failure	SIADH	Hyponatremia (116–130 mmol/L), serum hypoosmolarity (< 275mOsm/L) with normal dietary salt intake, increased urinary sodium, decreased daily diuresis (250–300 ml/day)
	Hypothyroidism	High TSH and low freeT4
	Disorders of calcium metabolism	Decreased calcium, vitamin D levels, recurrent spontaneous fractures
Gastrointestinal syndrome		Vomiting, diarrhea, glossitis, rectal prolapse, hematochezia, malabsorption syndrome, duodenitis with intestinal lymphangiectasia, catarrhal bauginitis and sigmoiditis (morphology and immunohistochemical staining)
Malabsorption		Deficiency of albumin, K, Fe, Zn, dyslipidemia
Arthritis, osteomyelitis		Arthritis of left hip and left knee joints, comminuted fractures of right and left femurs, compression fractures of Th 3–10, L4-5, left femur osteomielitis
Meningoencephalitis		Ophthalmoplegia, permanent muscular contractions, stammering, sleep disorders, recurrent attacks of aggression with arterial hypertension, pathological CSF findings (lymphocytic meningitis), MRI data correspond to presentation of encephalitis
Immunological disorders		Decreased CD3+, TH-cells, increased CD19+, abnormal ratio CD4+/CD8+, hyperexpression of general IgG, IgM and IgE, recurrent septic episodes

**Fig. 5:**
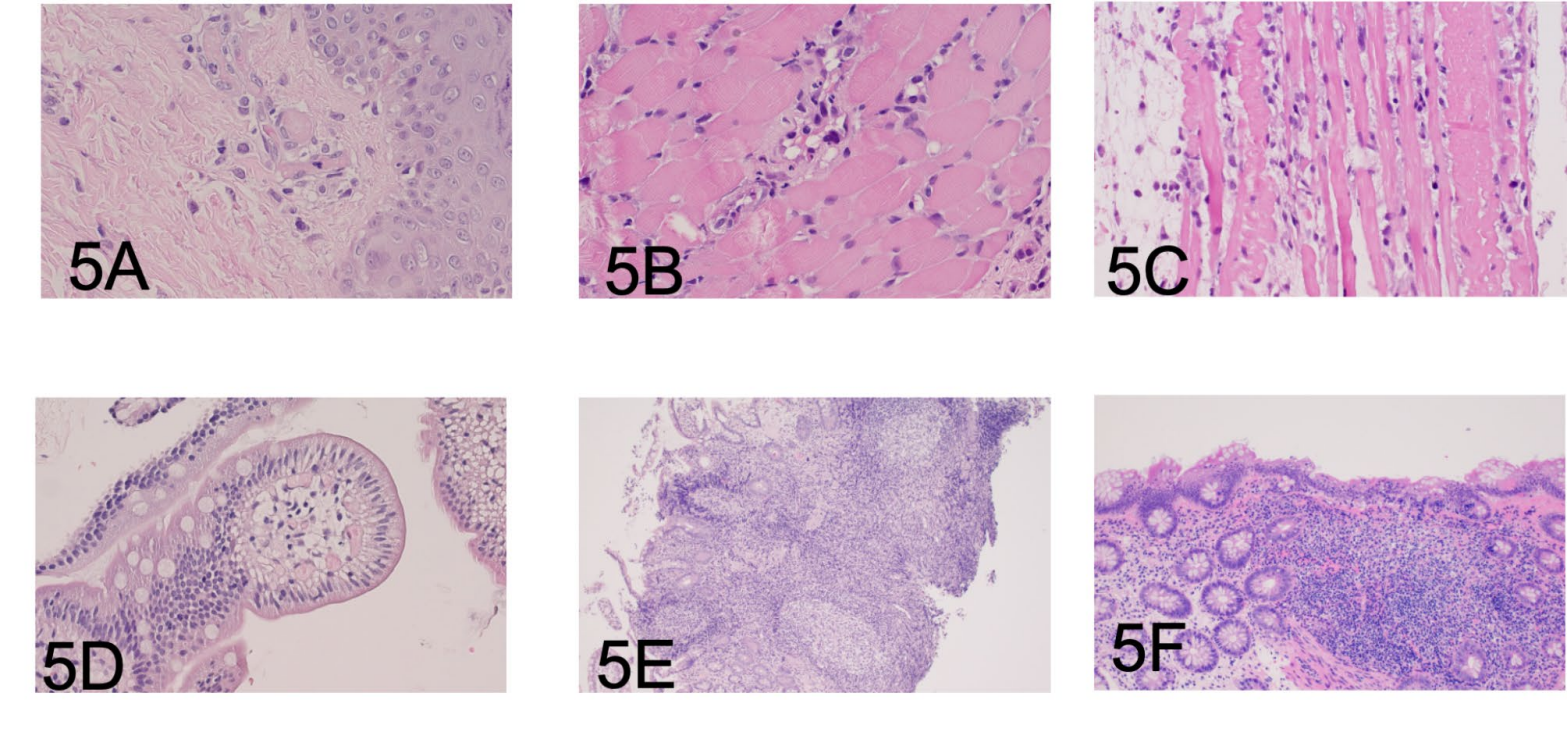
A. Skin. Endothelial cell swelling. Hematoxylin-eosin staining, ×60 B. Skeletal muscle vasculitis. Hematoxylin-eosin staining, ×60 C. Skeletal muscle myositis. Hematoxylin-eosin staining, ×60 D. Intestinal lymphangiectasia. Hematoxylin-eosin staining, ×40 E. Intestinal lymphofollicular hyperplasia. Hematoxylin-eosin staining, ×20 F. Lymphofollicular hyperplasia of colon. Signs of colitis Hematoxylin-eosin staining, ×20

Among all clinical syndromes the most unclear was SIADH, that was under control with tolvaptan therapy at the period of hospitalization to our clinic. According to literature search we met description of neurobrucellosis as one of possible causes of SIADH [10]. Taking in account anamnesis, clinical presentation, results of biopsy with signs of persistent infectious process (probably bacterial), follicular hyperplasia of lymph nodes, involvement of gastrointestinal tract and muscles (Fig. 5A F), we’ve decided to repeat tests for infectious diseases (CSF, blood, lymph nodes aspirates, bone marrow, saliva, urine and stool), using extended diagnostics with cultural, nucleic acid amplification methods (PCR) and serological tests. The results were negative for borreliosis, Whipple’s disease, helminthiasis, yersiniosis, pseudotuberculosis and Herpesviridae. CSF cytology revealed moderately elevated pleocytosis (8*10^6^/l) with predominance of lymphocytes (80%), increased protein (0,72 g/l). Repeated blood, urine and CSF cultures were negative. Serum tube agglutination test (SAT) for brucellosis was negative. Indirect fluorescent antibody test (IFA) for Brucella-specific antibodies in serum was positive for IgG – 4,86 (normal rage is < 1) and IgA – 1,4 (normal range < 1), and negative for IgM. PCR test of axillar lymph node aspirate was positive for *Brucella spp*.

**Fig. 6:**
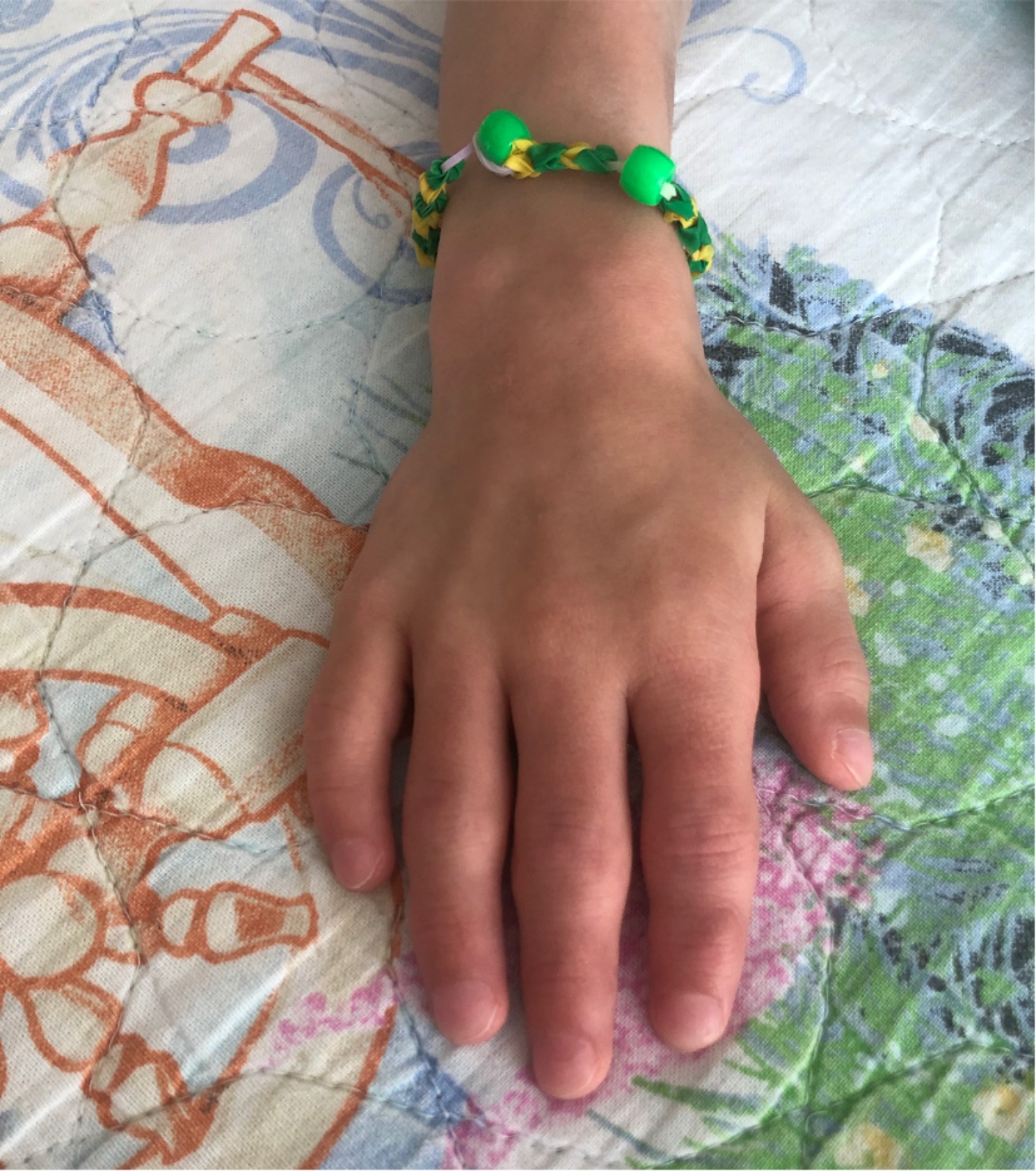
Resolution of hand dermatitis after 3 months of combined antibacterial therapy

**Fig. 7:**
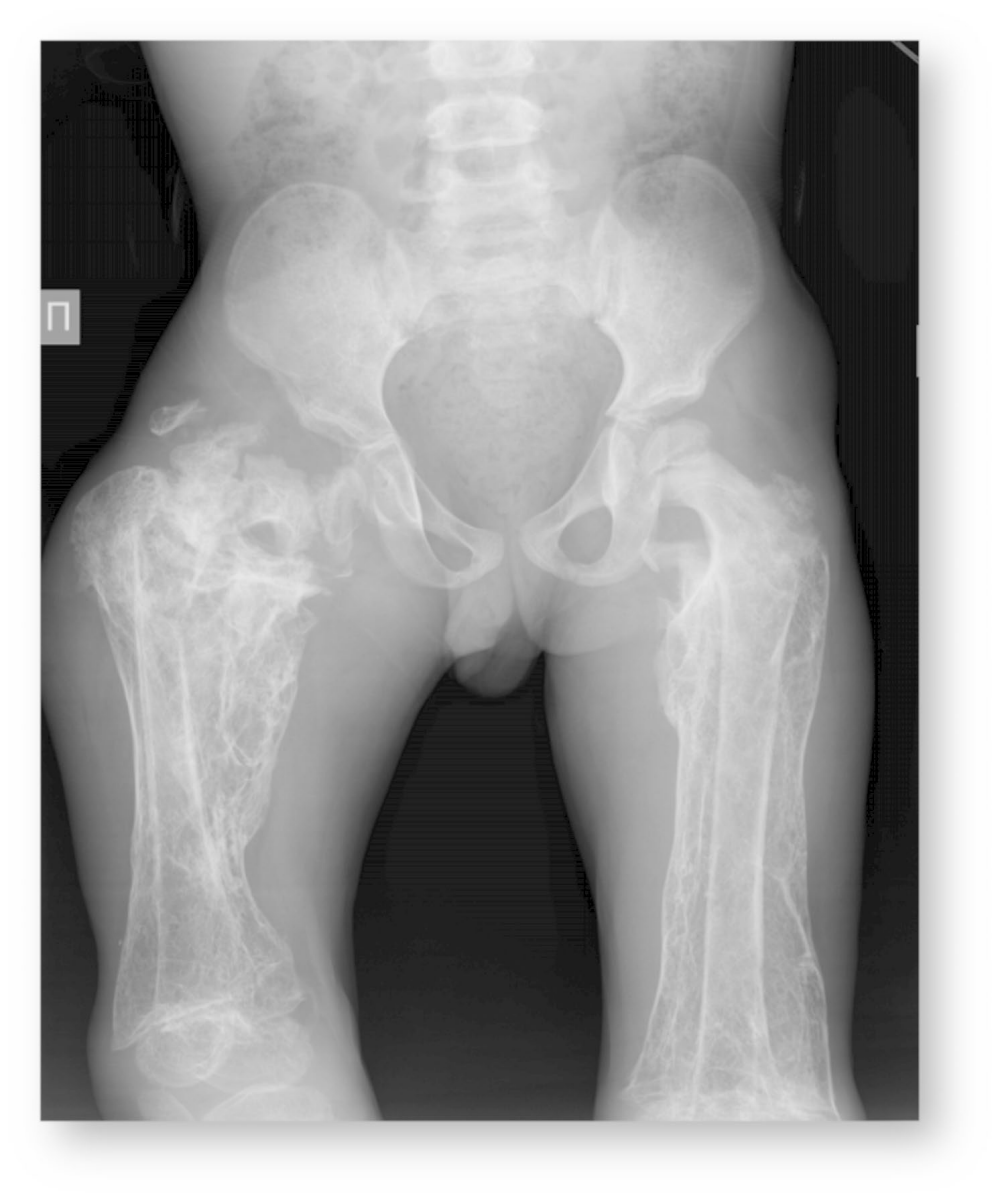
Resolution of joint contractures after 3 months of combined antibacterial therapy

In spite of lack of formal criteria of neurobrucellosis (positive culture of bacteria and serology in CSF) taking into account clinical and laboratory results, we confirmed chronic active phase of brucellosis with CNS involvement. Urgently combined antibacterial therapy was started according to guidelines for general pediatricians endorsed by the Saudi Pediatric Infectious Diseases Society (SPIDS) [4]. The recommended regime for neurobrucellosis in children < 8 years is rifampicin (20 mg/kg/day in two divided doses, max. 600 mg), trimethoprim/sulfamethoxazole (10 mg of trimethoprim/kg/day, max. 480 mg) and ciprofloxacin (30 mg/kg/day in two divided doses, max. 1,5 g) for 3–6 months up to one year in complicated cases [4]. Vitamin A was used as an immunomodulator supplementation. Concomitant to antibacterial therapy patient continued tolvaptan, antihypertensive and antiarrhythmic drugs, but 1 months later it became possible to start de-escalation of these therapy. To prevent progressive contractures of the lower extremities polymer corrective orthoses of the hip-foot were used. The effect was prompt, patient’s condition improved daily with full resolution of neurological and gastrointestinal symptoms, improvement of skin lesions (Fig. 6), normalization of laboratory abnormalities, recovery of motor activity and resolution of joint contractures (Fig. 7). A patient was followed-up closely in our clinic for 2 months and after discharge continued therapy at home with regular in-hospital check-ups (each 3–4 months). Due to severity of complications, osteomyelitis and protracted course of disease the duration of therapy was two years.

**Fig. 8:**
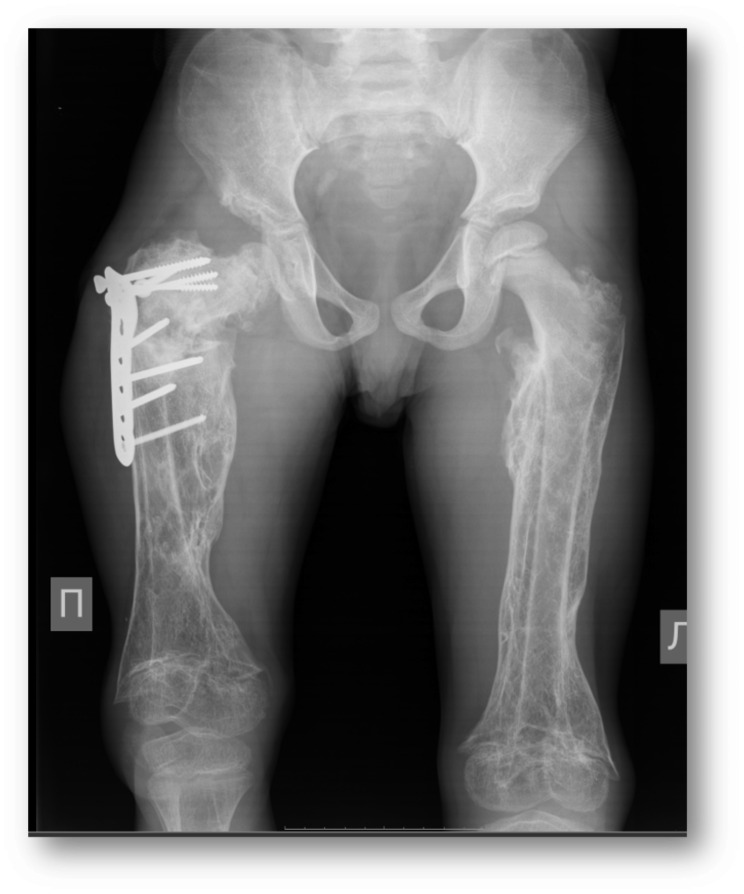
Repeated orthopedic surgery in order to improve limb deformities after femoral fractures and strengthen right hip joint

Later repeated orthopedic surgery was provided in order to improve lower limb deformities after femoral fractures and strengthen right hip joint (Fig. 8). The most severe changes were in the right hip and both knees which lead to the loss of movement and pathological position of the right leg (the combined flexion-rotation-adduction hip contracture of the flexion knee contracture) with complete impossibility for verticalization. The first surgery included hip reconstruction and the hyperostosis’ resection (it was at the age of 7 years and 1month, intraoperative bacteriological study was negative. Due to the very good clinical results – recovery of the hip and knee motions and active patient’s verticalization, - the second surgery was 9 months later to correct the left knee flexion contracture.

At the time of writing this report the patient has been under surveillance for 3 years (one and half year after first surgery) with no signs of active disease but with remain skeletal changes that require further observation and possible correction. Despite this, the patient has a sufficient range of movements in both hips and knees, and he can independently walk a long distance without any pain and serious restrictions.

## Discussion

Our case demonstrates brucellosis infection with a wide-range clinical spectrum and severe illness. According to literature reviews pediatric brucellosis usually presented in acute or subacute form [4, 5, 7, 10] with febrile illness (80–100%), constitutional symptoms (> 90%), joint involvement (55–80%), gastrointestinal (50%), hematological (20–40%) and neuropsychiatric (0,5–25%) disorders with rare progression to chronicity [2, 4, 11–15]. It should be underlined that childhood brucellosis usually produces mild to moderate disease and rarely progresses to chronic disease [2, 5]. Anamnesis of disease is one of the most important points in order to avoid such a delay in diagnosis.

We consider that our patient has a typical incubation period and disease presentation, developed 2–3 weeks after home-made dairies were used, but the absence of specific laboratory tests in acute phase could determine the subsequent diagnostic difficulties.

Despite variety of clinical signs in our patient, most of them were nonspecific and even with a huge number of different diagnostic methods the diagnosis couldn’t be verified for more than 18 months. One of unexplainable syndrome was SIADH which is a very rare disorder in pediatric population, but it is described in infections of the central nervous system like meningitis, encephalitis and abscess with an unclear mechanisms [8, 16]. The reported cases of hyponatremia and SIADH in children [8, 9, 17] and adults [16, 18] with neurobrucellosis helped us with further diagnostic route. Moreover, the presence of severe and continuous gastrointestinal involvement (morphologically identified), signs of hepatomegaly, splenomegaly, persistent peripheral lymphadenopathy, abnormalities in CSF (lymphocytic pleocytosis, increased protein and decreased glucose levels), findings on cranial MRI (signs of encephalitis) with described above clinical symptoms, relapsing episodes of osteomyelitis could correspond to chronic systemic inflammatory process.

The “golden standard” for verification of *Brucella* infection is isolation of *Brucella spp.* Culture and proceeding of bacterial colonies derived from blood, bone marrow, urine or CSF specimens require level 3 biosafety precaution protocols, long cultivation period (ranging from 4 to 7 days up to 45 days) and often demonstrate no bacterial growth, especially in chronic cases [5]. It should be noted that in chronic brucellosis standard cultural methods as well as serum agglutination tests such as Rose Bengal test (RBT) and standard agglutination test (SAT) can be uninformative [7, 19]. Therefore, a diagnosis of chronic form of disease is often based on clinical complaints along with results of more reliable serological testing such as enzyme-linked immunosorbent assay (ELISA) for detection of specific IgG and IgA and Coombs IgG test [19]. In order to verify the diagnosis, it is recommended to use all available methods together considering they different performance depending on a phase of disease [7]. In our patient standard agglutination test was repeatedly negative and we confirmed *Brucella* infection with ELISA (detection of specific IgG and IgA in serum), as well as PCR detection of *Brucella spp.* DNA in lymph node aspirate.

To our knowledge, there were no reported cases of pediatric Brucellosis with such a long and severe multiorgan involvement process. Taking in account rarity of some symptoms we present a literature review with pathophysiology of most impressive one.

Neurobrucellosis is serious complication of systemic brucellosis infection with a reported incidence as 0,5-25% 0–5,5% in the post antibiotic era [14, 20, 21]. Usually, neurobrucellosis is diagnosed in chronic stage of disease but it can be presenting manifestation. Meningitis or meningoencephalitis are the most common manifestations but symptoms could be nonspecific with headache and some behavioral and neuropsychiatric disorders abnormalities (sleeping disorders, epilepsy, agitation, muscular weakness, paresthesias et al.). CSF laboratory findings are not specific for neurobrucellosis, presented as elevated proteins, normal or low glucose, and a lymphocytic pleocytosis. Laboratory confirmation should be made with CSF cytology, serological and microbiological methods, thought the last one is rarely informative [20]. It is reported that diagnosis of neurobrucellosis is usually made 2–12 moths after the onset of symptoms in most cases that define long-term sequelae [14]. Simultaneously or independently of neurobrucellosis endocrine disorders may occur. SIADH in patients with brucellosis was revealed with variable frequency and an unclear etiology [9, 17]. Possible elucidation is ectopic ADH secretion and SIADH as a result of direct involvement of CNS, ADH stimulation by proinflammatory cytokines (Il-6) or in case of stress/ nausea and vomiting that accompany infections [9]. Symptomatic long-lasting hyponatriemia is strongly associated with mortality mainly due to cerebral edema in case of inappropriate treatment modalities. Vasopressin receptor agonists are shown to be effective and well tolerated during correction of hyponatremia nonresponding to fluid restriction and sodium intake [8, 16]. In our patient tolvaptan demonstrated good efficacy for sodium level control until beneficial effect of antibiotic treatment was achieved. Moreover, other metabolic abnormalities such as increased excretion of catecholamine’s derivatives, hypothyroidism and dyslipidemia (high levels of cholesterol and triglyceride, lower levels of LDL and HDL) were observed. The literature contains limited information about patterns of involvement, but due the fact that endocrine organs are highly vascularized they are expected to harbor the bacterium as a secondary localization [22].

Diverse gastrointestinal manifestations in brucellosis, ranged from moderate complaints like anorexia (22–45%), vomiting (7–18%), diarrhea (3–6%), mesenteric lymphadenitis to serious complications like liver (32–63,3%) or spleen (29–56,6%) involvement and rare life-threatening symptoms as cholecystitis, colitis, pancreatitis, peritonitis, intestinal obstruction [23]. Anorexia and vomiting are likely to be associated with central effect of inflammatory cytokines like INF gamma and TNF-α [23]. The histological examination of colon wall reveals acute or chronic inflammatory process with mucosal effacement, extensive infiltration with lymphocytes and plasma cells along with macrophages and some neutrophils in lamina propria [23, 24].

One of the most impressive symptoms in our patient was severe skin involvement on both hands. We didn’t find similar cases in literature, indeed cutaneous lesions are rare in Brucellosis [25]. The main pathogenic mechanisms include direct inoculation of the *Brucella spp*. into the skin, hypersensitivity phenomena, deposition of immune complexes in the skin and invasion of the skin via a hematogenous route of spreading [25]. We proposed that skin lesion in our patient could be, at some extend, associated with severe Zinc deficiency.

Osteoarticular complications are most frequent with 10–85% infected patients affected [26]. The most common forms are sacroiliitis (up to 80%), spondylitis and spondylodiscitis (up to 54%), with the lower prevalence of peripheral arthritis, osteomyelitis, discitis, bursitis and tenosynovitis [26].

Moreover, *Brucella* bacteria have a tropism for osteoarticular localization and may cause bone destruction, osteopenia and cartilage damage [22]. The main mechanisms are associated with invading and replication within human osteoblasts, inhibiting their differentiation, mineral and organic matrix deposition and inducing apoptosis. Additionally excessive production of proinflammatory cytokines, chemokines and matrix metalloproteases by infected macrophages, induce osteoclasts number and its activity (defined as pathological osteoclastogenesis), resulting in excessive bone resorption and destruction [22]. Besides this, Kurtaran et al. confirmed the significantly lower vitamin D and soluble vitamin D receptor levels in brucellosis patients in all age groups compared with a healthy population [13]. Both facts can explain bone damage and recurrent fractures in our patient. Antibiotics and surgery are the only two options for the treatment and cure of osteoarticular brucellosis [26].

Hematological manifestations of brucellosis are well described (anemia-64%, thrombocytopenia-28%, leucopenia-38%, pancytopenia – 2–14%). Several possible mechanisms are proposed such as hypersplenism, granuloma formation in the bone marrow, phagocytosis of formed elements by reticuloendothelial cells or bone marrow depression due to associated septicemia [15]. Although anemia in brucellosis is related to bone marrow involvement, autoimmune processes, malabsorption syndrome are also reported as pathogenetic mechanisms [15, 27].

Immunological disfunction in patients with brucellosis present a great interest and are differ in cases of acute and chronic forms. The decline of T-helper and cytotoxic T-lymphocytes together with activation of humoral immunity and increasing of B-lymphocytes, levels of immunoglobulins A and G have been described in literature reports and were revealed in our case [9, 16, 28]. Most studies of the Ig isotype response in brucellosis concerned IgG, IgM and IgA, mostly, for differentiating between the acute and chronic stages of disease [29]. Besides these classes of Ig elevation, IgE was found to be a part of immune response and a helpful marker in post treatment monitoring and predicting a risk of relapse in both groups [30, 31]. Mentioned data indicate on the formation of secondary immunodeficiency affecting the progress of brucellosis and evidence of necessity for immune regulation therapy [28, 32].

Concerning specific histologic patterns of involved tissues, the common sign for infected tissues by brucellosis would be chronic inflammation, characterized by lymphocytic infiltration with variable numbers of histiocytes and plasma cells. Granulomatous inflammation, acute necrotizing and suppurative inflammation are less common [27, 30]. The identical changes we revealed in biopsied tissues (bones, intestine, lymph node, skin) of our patient with predominance of lymphoid tissue hyperplasia, infiltration of macrophages and lymphocyte and vasculitis phenomenon.

Adequate antibiotic therapy can ameliorate all associated complications of brucellosis, even, in severe cases [1, 2, 4, 5, 7–11, 18]. In our patient we demonstrate the perfect response to triple antimicrobial regimen with almost complete recovery, except bone deformations which require further monitoring and surgical correction. Also, we have to continue hormone replacement therapy in order to control hypothyroidism, but in significantly lower doses. It should be noted that combined antibacterial therapy in our patient was well tolerated and we didn’t register any toxicity.

## Conclusion

Our clinical case demonstrates severe multisystem manifestation of chronic brucellosis. Doctors of all specialties should be aware of clinical signs of brucellosis and keep in mind the necessity of collecting anamnesis related to zoonotic infections and diverse results of serological tests for brucellosis depending on duration of disease. SIADH could be one of the most important symptoms for diagnosing brucellosis. Multidisciplinary approach is crucial for appropriate management of chronic stage brucellosis.

## Electronic supplementary material

Below is the link to the electronic supplementary material.

Supplementary Material 1

Supplementary Material 2

## Data Availability

The datasets used and/or analysed during the current study available from the corresponding author on reasonable request.
