# Supplementary material for: Presentation of severe brucellosis in 5-year-old boy - challenges and results
Source: BMC Infect Dis. 2023 Mar 17;23:166. doi: 10.1186/s12879-023-08138-7 (PMC10022032; doi:10.1186/s12879-023-08138-7)
Supplement: Supplementary file 2 — Supplementary Material 2 [file 12879_2023_8138_MOESM2_ESM.docx]

**Supplementary material legend:**

**Video 1.** Video of chaotic spontaneous muscular contractions of thigh
